# Small molecule NSC1892 targets the CUL4A/4B-DDB1 interactions and causes impairment of CRL4^DCAF4^ E3 ligases to inhibit colorectal cancer cell growth

**DOI:** 10.7150/ijbs.40235

**Published:** 2020-02-04

**Authors:** Chunmei Yang, Jing Wu, Hongbo He, Hong Liu

**Affiliations:** 1 Department of Integrated Traditional and Western Medicine, Chengdu Shangjinnanfu Hospital/West China Hospital of Sichuan University, Chengdu 610041, Sichuan, China; 2 Department of Integrated Traditional and Western Medicine, West China Hospital of Sichuan University, Chengdu 610041, Sichuan, China

**Keywords:** NSC1892, CUL4A, CUL4B, DDB1, ST7, colorectal cancer

## Abstract

Cullin 4A and 4B (CUL4A and 4B) function as oncogenes in colorectal cancer (CRC) cells. Both of them conservatively associate with DNA damage-binding protein 1 (DDB1) and DDB1-CUL4-associated factor 4 (DCAF4) to form Cullin-RING E3 ligases known as CRL4^DCAF4^, which specifically ubiquitinate and degrade tumor suppressor ST7 (suppression of tumorigenicity 7). Knockdown either *CUL4A/4B* or *DDB1* significantly inhibits tumor cell growth *in vitro* and *in vivo*. Thus, targeting these CRL4^DCAF4^ components and their interactions may be an effective strategy for the therapy of CRC. In this study, we developed an *in vitro* AlphaScreen assay to identify small molecules targeting the CUL4A-DDB1 interaction. We obtained a compound NSC1892, which strongly disrupted the CUL4A-DDB1 interaction (IC_50_ = 1.8 μM). Oncogenic phenotype analyses indicated that NSC1892 showed significant cytotoxicity to decrease cell proliferation, colony formation and invasion in CRC cells. Biochemical analyses demonstrated that NSC1892 treatment did not change CUL4A and CUL4B protein levels, but caused the degradation of DDB1, thereby leading to the impaired assembly of CRL4^DCAF4^ E3 ligases and resulting in the accumulation of ST7. The administration of NSC1892 in mice also significantly inhibited tumor growth through degrading DDB1 and accumulating ST7. Interestingly, NSC1892 also showed promising cytotoxicity to decrease the growth of other *CUL4A-* or *CUL4B-*overexpressing tumor cells such as SKOV3 ovarian cells and Saos2 osteosarcoma cells. Our results provide a new avenue for the development of a therapeutic compound targeting tumors through disrupting the CUL4-DDB1 interaction.

## Introduction

Colorectal cancer (CRC) is one of the ubiquitous and high-incidence of cancers in the world 1-3. The pathological mechanisms of CRC are very complicated and mainly include genetic instability (e.g., mutations of tumor suppressors and cell cycle regulators), microenvironment changes, overexpression of oncogenes, and aberrant expression of non-coding RNAs 4-6. Like many other tumors, treatment for CRC usually involves surgery, radiation therapy and chemotherapy 1-6. In recent several years, personalized medicines are developing rapidly in the treatment of CRC 1, 7. For instance, the abnormality of epidermal growth factor receptor (EGFR) is an important cause that contributes to the development and growth of CRC 1, 7. Two anti-EGFR monoclonal antibodies cetuximab and panitumumab have been utilized in the personalized treatment of CRC patients with EGFR overexpression 1, 7. In addition, bevacizumab can specifically target vascular endothelial growth factor (VEGF) to prevent the growth of blood vessels in tumors 1, 7. However, there is still no personalized medicine for many other differentially expressed genes involved in the pathogenesis of CRC 1, 7.

Ubiquitination is one of the major protein modifications, and it is driven by a cascade including ubiquitin-activating enzymes (E1s), ubiquitin-conjugating enzymes (E2s), and ubiquitin ligases (E3s) 8, 9. Currently, more than 600 E3s have been identified in the human genome 10. Of them, Cullin-RING ubiquitin ligases (CRLs) represent the largest subfamily and they harbor more than 400 members 11. Commonly, a CRL complex consists of four different components including a cullin protein (CUL1, -2, -3, -4A, -4B, -5, -7 or -9 in human), a RING box protein (RBX1 or -2), an adaptor protein [S-phase kinase-associated protein 1 (Skp1), Elongin B and C, or damaged DNA binding protein 1 (DDB1)], and a substrate recognition receptor [F-box proteins, SOCS (suppressor of cytokine signaling proteins), BTB (broad complex, tramtrack, bric-a-brac) proteins or DCAF (DDB1 and CUL4-associated factors)] 10-13. CRLs especially CRL4s are often aberrantly activated in multiple human cancers such as CRC 14, breast cancer 15, non-small-cell lung cancer (NSCLC) 16, ovarian cancer 17, osteosarcoma 18, and squamous cell carcinoma 19. The human CUL4A and CUL4B share over 80% amino acid sequence identity and both of them assemble a complex with DDB1, RBXs and DCAFs 14-19. However, they do not show significantly functional redundancy in most majority of cancer types 14-19. Recently, we have revealed that both CUL4A and CUL4B associate with RBX1, DDB1, and DCAF4 to assemble two separate CRL4^DCAF4^ E3 complexes, whose activation causes the hyper-ubiquitination and degradation of ST7 (suppression of tumorigenicity 7), eventually leading to the pathogenesis of CRC 14. Specific knockdown of *CUL4A*, *CUL4B* or *DDB1* markedly decreases cancer cell growth 14. These results together with the conserved interactions of CUL4A/4B-DDB1 and CUL4A/4B-RBX1 encourage us to screen small molecules using these individual molecules or their interactions as targets.

In recent years, a highly sensitive method known as AlphaScreen has been widely used to obtain compounds that target the protein-protein interactions 20-23. The principle of AlphaScreen is based on two protein interactions, which brings their associated “Donor” and “Acceptor” beads together 20-23. After laser excitation at 680 nm, a photosensitizer located in the “Donor” beads converts O_2_ to an excited state ^1^O_2_, which activates fluorophores located in the “Acceptor” beads 20-23. The emission of fluorophores can be detected at 520-620 nm. Small molecules that disrupt two protein interaction can decrease the intensity of chemiluminescence 20-23 To identify compounds that disrupt CRL4^DCAF4^ E3 ligase, we developed an *in vitro* AlphaScreen high throughput screening (HTS) assay using the CUL4A-DDB1 interaction as a target. Using this method, we discovered NSC1892 showed a strong ability to inhibit CUL4A-DDB1 interaction. We then evaluated the cytotoxic effect of this compound on the growth of CRC cells and measured molecular changes of CRL4^DCAF4^ complexes after NSC1892 treatment. Our *in vitro* and *in vivo* data suggest that NSC1892 is an effective compound inhibiting CRC cell growth through impairing the assembly of CRL4^DCAF4^ E3 ligases.

## Materials and methods

### Protein purification

The coding regions of *DDB1* and *CUL4A* cDNAs were cloned into the pET28a (His tag) and pGEX-6P-1 (GST tag) vectors between BamHI and EcoRI sites, respectively. The pET28a-DDB1 and pGEX-6P-1-CUL4A plasmids were transformed into an* Escherichia coli* strain BL21 (DE3.0), respectively. The positive colonies were grown in liquid lysogeny broth (LB) medium containing antibiotics to the logarithmic phase. Cells were then induced with 1 mM isopropyl β-D-thiogalactoside (IPTG) for 12 h at 16 °C. Cells expressing GST-CUL4A were lysed in a buffer containing 1×PBS, 1 mM DTT, 0.01% Tween-20, and 1 mM PMSF. GST-CUL4A protein was purified using Glutathione Sepharose 4B resin (GE Healthcare, Chicago, IL, USA, #GE17-0756-01). Cells expressing His-DDB1 were lysed in a buffer containing 50 mM NaH_2_PO_4_, 300 mM NaCl, 10 mM imidazole, 1 mM DTT, 0.01% Tween-20, and 1 mM PMSF. His-DDB1 protein was purified using Ni-NTA resin (ThermoFisher Scientific, Waltham, MA, USA, #88221). Both purified proteins were stored at -80 °C until use.

### Small molecule screening

A naturally sourced small molecule pool was composed of 2000 compounds, which were isolated from plants and determined by the nuclear magnetic response (NMR) spectroscopy. Small molecules disrupting the CUL4A-DDB1 interaction were screened using an AlphaScreen detection kit (PerkinElmer, Waltham, MA, USA, #6760603M) following a protocol provided by the manufacturer. Briefly, 100 nM of each protein was combined with 10 μL of AlphaScreen donor and acceptor beads, respectively. The protein-binding beads were then incubated in an assay buffer containing 50 mM Tris (pH 8.0), 100 mM NaCl, 0.03% BSA and 0.01% Tween-20), followed by adding 5 μM individual small molecule into each well. After incubation at 25 °C for 2 hrs, the 384-well assay plates (PerkinElmer, #6008350) were read in an Envision Multilabel Reader (PerkinElmer, #2105-0010). Small molecules that decreased AlphaScreen signal (<5000) were selected as candidates.

### Cell lines, cell culture and transfection

Two CRC cell line HCT-116 (#CCL-247) and HT-29 (#HTB-38), one osteosarcoma cell line Saos2 (#HTB-85), one ovarian cancer cell line SKOV3 (#HTB-77), and one noncancerous osteoblast cell line hFOB1.19 (#CRL-11372) were obtained from the American Type Culture Collection (ATCC) (Manassas, VA, USA). One human colon epithelial cell line (HCEC-1CT) was obtained from Evercyte (Vienna, Austria). HCT-116, HT-29, Saos2, and SKOV3 cells were cultured in ATCC-formulated McCoy's 5a Medium (#30-2007) supplemented with 10% heat-inactivated fetal bovine serum (FBS) (Sigma, St. Louis, MO, USA, #F2442) and 50 U/mL penicillin-streptomycin (PS) (Sigma, #P4333). The growth medium and conditions were the same as described previously 14. hFOB1.19 cells were incubated in F12/DMEM (ThermoFisher Scientific, #12660012) containing 10% FBS, 50 U/mL PS and 2.5 mM L-glutamine (ThermoFisher Scientific, #25030081). hFOB1.19 cells were grown at 34°C, and the other cells were incubated at 37°C. Cell transfection with plasmids and siRNA was performed using a Lipofectamine 2000 reagent (Thermo Fisher Scientific, #11668019) following a previous protocol 14. Briefly, cells (1×10^6^) were transfected with 100 ng plasmids (pCDNA3-2×Flag-ST7, pCDNA3-3×HA-Ubiquitin, and pCDNA3-3×HA-Ubiquitin-K48R) or 5 pmol DDB1-siRNA (Sigma, #SASI_Hs01_00242315) in combination with lipofectamine 2000. After culturing at 37°C for another 48 hrs, cells were subjected to extract RNA and protein.

### Cell proliferation assay

HCT-116, HT-29, Saos2, SKOV3, HCEC-1CT, and hFOB1.19 cells under 80% confluence were treated with DMSO, 0.2 μM, 2.0 μM and 20 μM NSC1892, respectively. Cell viability was examined at 4 h (0 day), 1, 2, 3, 4 and 5 days using an MTT kit (Sigma, #11465007001) following the manufacturer's guidelines.

### Colony formation and cell invasion assays

HCT-116 cells were seeded in 6‐well plates (ThermoFisher Scientific, #140675) with a density of 500 cells per well and then were continuously grown in serum‐free ATCC-formulated McCoy's 5a medium supplemented with DMSO, 0.2 μM, 2.0 μM and 20 μM NSC1892, respectively. Cells were grown at 37°C for two weeks with a medium change every three days. Cell colonies were fixed with 1 × PBS buffer containing 4% paraformaldehyde (Sigma, #252549) for 15 min and stained with 0.2% crystal violet (Sigma, #C0775) for 20 min at room temperature. Colony numbers were counted using Image J software. For cell invasion assay, a total of 1 × 10^5^ HCT-116 cells in serum-free ATCC-formulated McCoy's 5a Medium were seeded into the upper chamber of Boyden chambers, followed by adding DMSO, 0.2 μM, 2.0 μM or 20 μM NSC1892 into cell suspension. The lower chambers were filled with ATCC-formulated McCoy's 5a Medium containing 10% FBS. After incubation 37°C for 24 hrs, cells in the upper layer were removed with cotton wool, and cells on the surface of lower chambers were fixed with methanol for 30 min and stained with 0.2% crystal violet for 20 min at room temperature. Cells were then photographed using an IX71 microscope (Olympus, Tokyo, Japan).

### Immunoprecipitation and immunoblots

Cultured cells and tumor tissues were lysed in RIPA buffer (ThermoFisher Scientific, #89901) containing 1 × complete protease inhibitor cocktail (ThermoFisher Scientific, #78425). For immunoprecipitation assay, cell lysates were incubated with protein A agarose (Abcam, Cambridge, MA, USA, #ab193254) and anti-DDB1 (Abcam, #ab9194) or anti-CUL4A (Abcam, #ab92554). The purified DDB1-complex and CUL4A-complex were subjected to examine protein levels of CRL4^DCAF4^ members using anti-CUL4A, anti-CUL4B (Abcam, #ab67035), anti-RBX1 (Sigma, #SAB1410048), anti-DDB1, and anti-DCAF4 (Abcam, #ab83655). For western blotting assay, equal amounts of cell lysates or purified proteins were loaded into 12% SDS-PAGE gels for separation. Except for the above antibodies, primary antibodies used in immunoblot assays included anti-c-Myc (Abcam, #ab39688), anti-ST7 (Abcam, #ab122460), and anti-GAPDH (Abcam, #ab8245). The protein signals were visualized using an ECL kit (Thermo Fisher Scientific, #32106) and recorded in a ChemiDoc MP instrument (Bio-Rad Laboratories, Hercules, CA, USA, #17001402).

### RNA extraction and qRT-PCR analysis

Total RNA was extracted from cultured cells following a previous protocol 14. Briefly, 1 × 10^6^ cells were lysed in 500 μL TRIzol (Thermo Fisher Scientific, #15596026), followed by adding 200 μL chloroform (Sigma, #650498). After centrifuging at 13,000 rpm for 15 min, the supernatant was precipitated with 100% ethanol overnight. To synthesize the first-strand cDNA, 1 μg RNA of each sample was reacted with an M-MuLV reverse transcriptase kit (New England Biolabs, Ipswich, USA, #M0253S). The single-stranded cDNA was diluted 20-fold and the mRNA levels of *c-MYC*, *CUL4A*, *CUL4B*, *DDB1*, and *ST7* were detected by qRT-PCR analyses using an SYBR Green kit (Thermo Fisher Scientific, #A25780) on a Bio-Rad CFX96 real-time PCR detection system with primers listed in Supplementary Table-1. β-actin was used as an internal control to normalize the individual gene expression levels.

### *In vitro* pulldown assay

Equal amounts (2 μg) of GST-CUL4A and His-DDB1 proteins were mixed to form the CUL4A-DDB1 complex at 4°C for 30 min. The CUL4A-DDB1 complex was then treated with DMSO, 0.2 μM, 2.0 μM or 20 μM NSC1892 at 4°C for 6 hrs, followed by incubating with the Ni-NTA resin (Sigma, #70666-3) at 4°C for 4 hrs. The Ni-binding proteins were washed with a buffer containing 50 mM Tris (pH 8.0), 75 mM NaCl, 75 mM imidazole, and 1 mM DTT. The pulldown proteins were determined by loading into a 10% SDS-PAGE gel and then stained with Coomassie blue.

### Ubiquitination assay

The ubiquitination assays were carried out as described previously 14. For *in vitro* ubiquitination assay, the His-ST7 protein purified from *Escherichia coli* and Flag-CUL4A complex purified from HCEC-1CT cells were mixed with 0.1 μM E1 (Sigma, #U5633), 0.4 μM E2 (Sigma, #662098), and different concentrations of NSC1892 (0, 2 and 20 μM) for 75 min. The ubiquitination of ST7 was examined by western blotting with an anti-ubiquitin antibody. For *in vivo* ubiquitination assay, HCEC-1CT cells were co-transfected with pCDNA3-2×Flag-ST7 and HA-ubiquitin, followed by incubation at 37°C for 48 hrs. The resulting cells were further treated with DMSO, 0.2 μM, 2.0 μM or 20 μM NSC1892 for 6 hrs, and then were lysed in buffer containing 150 mM NaCl, 1% SDS, 10 mM Tris-HCl (pH 8.0), and 1 × complete protease inhibitor cocktail. The anti-Flag-agarose resin (Sigma, #F2426) was used to purify the Flag-ST7-associated protein complex. Equal amounts of proteins were resolved in 12% SDS-PAGE gel and ST7 ubiquitination was detected using an anti-HA antibody.

### *In vivo* tumor growth inhibition assay

Athymic *nu/nu* mice (Shanghai SLAC Laboratory Animal Co. Ltd., Shanghai, China) were maintained following the guidelines approved by the Institutional Animal Care and Use Committee (IACUC) of Sichuan University. HCT-116 cells (5 × 10^6^) were suspended in 100 μL of a mixture of PBS and Matrigel (1:1 ratio, v/v) (BD Biosciences, San Jose, CA, USA, #354234), followed by injecting subcutaneously into the flanks of 6-week-old female mice. Tumors were assessed and measured with fine calipers at 5-day intervals, and tumor volumes were calculated with the formula: Volume* =* (Length* ×* Width^2^)/2. After 25 days, mice harboring ~400 mm^3^ tumor volumes were randomly assigned to four groups (*n* = 5). The first group was set as control and only injected with DMSO. The other three groups were injected with 0.2 μM, 2.0 μM or 20 μM NSC1892 twice a week, respectively. Tumors were also assessed and measured with fine calipers at 5-day intervals for another 40 days.

### Statistical analysis

Values were the mean ± standard deviation (SD) of at least three independent experiments. The statistical analysis of the data was performed using a two-sided Student's *t* test. Significance was set at *P* < 0.05 (*), *P* < 0.01 (**) and *P* < 0.001 (***). Figures were prepared using the Prism GraphPad (version 8).

## Results

### Small molecule NSC1892 specifically repressed the CUL4A-DDB1 interaction

Our recent publication showed that both CUL4A and CUL4B were significantly overexpressed in CRC cells and tumors. Both of these two proteins directly interacted with DDB1, and the formed complexes acted as scaffolds to assemble CRL4^DCAF4^ E3 ligases 14. Specific knockdown of *CUL4A*, *CUL4B* or *DDB1* resulted in tumor cell growth inhibition *in vivo* and *in vitro* 14. To validate this observation, we carried out an independent experiment to knock down *CUL4A*, *CUL4B* or *DDB1* and evaluate cell growth inhibition. Consistent with previous results, we also observed a significant growth inhibition after their knockdown (Supplementary Figure 1). These results suggested that CUL4A/4B and DDB1 were promising candidate targets for the treatment of CRC tumor. However, there are still no commercial medicines targeting any of them.

**Figure 1:**
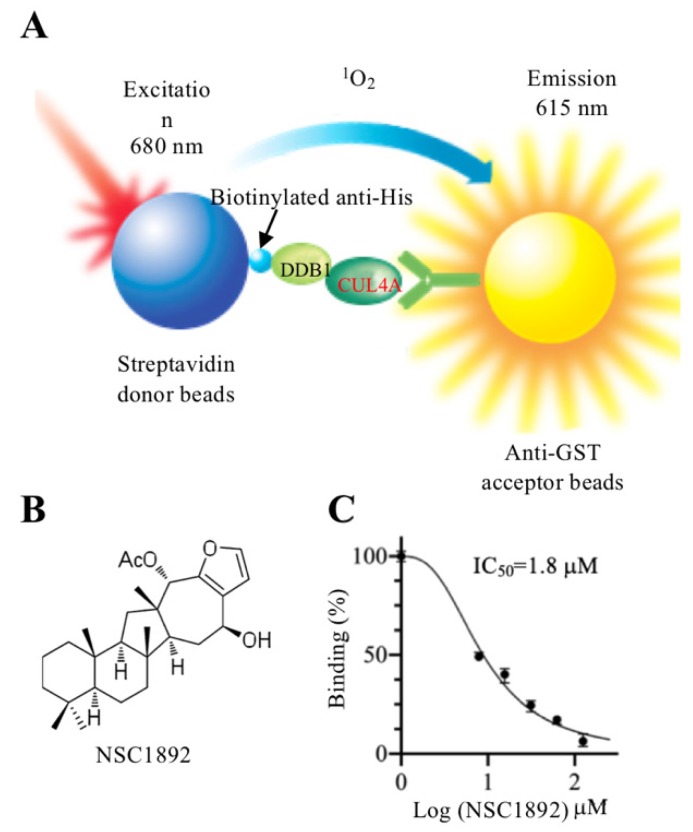
** NSC1892 specifically disrupted the CUL4A-DDB1 interaction in an AlphaScreen system. (A)** A schematic diagram of *in vitro* AlphaScreen system. The purified His-DDB1 was biotinylated and associated with the Streptavidin-coated donor beads, and GST-CUL4A was associated with the anti-GST acceptor beads. Upon illumination at 680 nm, donor beads converted ambient oxygen to ^1^O2. The energy was transferred to acceptor beads and subsequently culminated in light production at 615 nm. **(B)** The chemical structure of NSC1892. **(C)** NSC1892 inhibited the CUL4A-DDB1 interaction with an IC_50_ of 1.8 μM. A secondary AlphaScreen assay was performed to determine the inhibitory effect of NSC1892 on the CUL4A-DDB1 interaction.

To identify compounds that specifically target CRL4^DCAF4^ complexes, we next determined to screen small molecules disrupting the CUL4A-DDB1 interaction. For this purpose, we developed an *in vitro* AlphaScreen HTS assay, which has been successfully utilized to screen compounds inhibiting protein-protein interactions in many groups 20-23. DDB1 was constructed into a pET28a vector and CUL4A was cloned into a pGEX-6P-1 vector. After obtaining good quality of recombinant proteins (Supplementary Figure 2), the His-DDB1 and GST-CUL4A proteins were bound to streptavidin donor beads and GSH-linked donor beads, respectively (Figure 1A). After setting a reaction by adding individual compound into a mixture containing 100 nM of His-DDB1 and GST-CUL4A proteins and 10 μL of AlphaScreen donor and acceptor beads, we screened a total of 2,000 compounds and obtained four compounds (NSC0623, NSC0985, NSC1268 and NSC1892) that showed different abilities to inhibit the CUL4A-DDB1 interaction (Figures 1B and 1C and Supplementary Figure 3). Among these compounds, NSC1892 (Figure 1B) exhibited the strongest ability to inhibit CUL4A-DDB1 interaction with an IC_50_=1.8 μM (Figure 1C), while the IC_50_ of NSC0623, NSC0985, NSC1268 were 12.9 μM, 25.3 μM and 34.2 μM, respectively (Supplementary Figure 3). Thus, we will focus our following studies on evaluating the effect of NSC1892 on tumor cell growth inhibition and revealing the underlying molecular changes after NSC1892 treatment.

### NSC1892 markedly inhibited CRC cell growth *in vitro*

**Figure 2:**
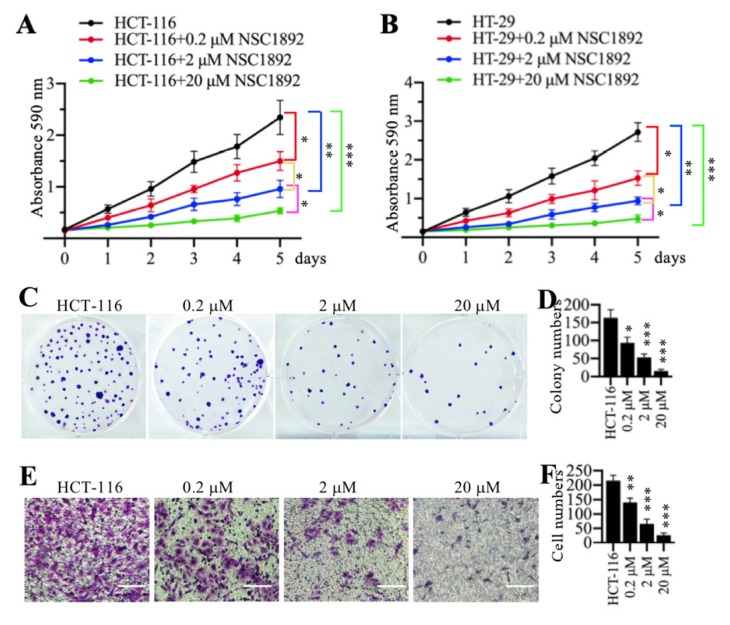
** NSC1892 inhibited oncogenic phenotypes *in vitro*. (A** and** B)** NSC1892 treatment inhibited cell proliferation. HCT-116 **(A)** and HT-29 **(B)** cells were treated with different concentrations (0, 0.2, 2, and 20 μM) of NSC1892 for 5 days. Cells viability was measured each day using an MTT assay. ^*^P <0.05, ^**^P <0.01 and ^***^P <0.001. **(C** and** D)** NSC1892 treatment decreased colony formation. HCT-116 cells were seeded into a six-well plate with a density of 500 cells per well, followed by adding DMSO, 0.2 μM, 2.0 μM and 20 μM NSC1892 into medium and continuously growing for two weeks. Colonies were fixed with 4% paraformaldehyde and stained with 0.2% crystal violet **(C)**. Colony numbers were counted with Image J software **(D)**. ^**^P <0.01 and ^***^P <0.001.** (E** and** F)** NSC1892 inhibited cell invasion. HCT-116 (1 × 10^5^) cells were seeded into the upper chamber of Boyden chambers, followed by adding DMSO, 0.2 μM, 2.0 μM or 20 μM NSC1892 into cell suspension. After 24 hrs, cells in the lower chambers were fixed in methanol and stained with 0.2% crystal violet **(E)**. Scale bars=100 μm. cell numbers were quantified **(F)**. ^**^P <0.01 and ***P < 0.001.

Since NSC1892 was able to disrupt the CUL4A-DDB1 interaction *in vitro*, we next sought to determine its effect on tumor cell growth. Accordingly, we treated two CRC cell lines HCT-116 and HT-29 with 0, 0.2, 2 and 20 μM NSC1892, respectively. Cell proliferation assay results indicated that NSC1892 inhibited cell growth in a dose-dependent manner (Figures 2A and 2B). In a lower dose (0.2 μM), NSC1892 treatment resulted in ~40% reduction of cell proliferation at the 5-day time point (Figures 2A and 2B). In contrast, 2 and 20 μM NSC1892 treatments caused ~60% and ~80% decrease, respectively (Figures 2A and 2B). In addition, we also evaluated colony formation and cell invasion abilities using the same concentrations of NSC1892. The colony formation assay results indicated that 0.2, 2 and 20 μM NSC1892 treatments resulted in a ~40%, ~66% and ~85% reduction in colony numbers compared to control (Figures 2C and 2D). The cell invasion results showed that the invading cell numbers significantly decreased with the increase of NSC1892 concentrations (Figures 2E and 2F). Treatments with 0.2, 2 and 20 μM NSC1892 caused ~36%, ~65% and ~90% reduction, respectively (Figures 2E and 2F). These results suggested that NSC1892 had strong cytotoxicity to inhibit CRC cell growth *in vitro*.

### NSC1892 treatment caused the degradation of DDB1

**Figure 3:**
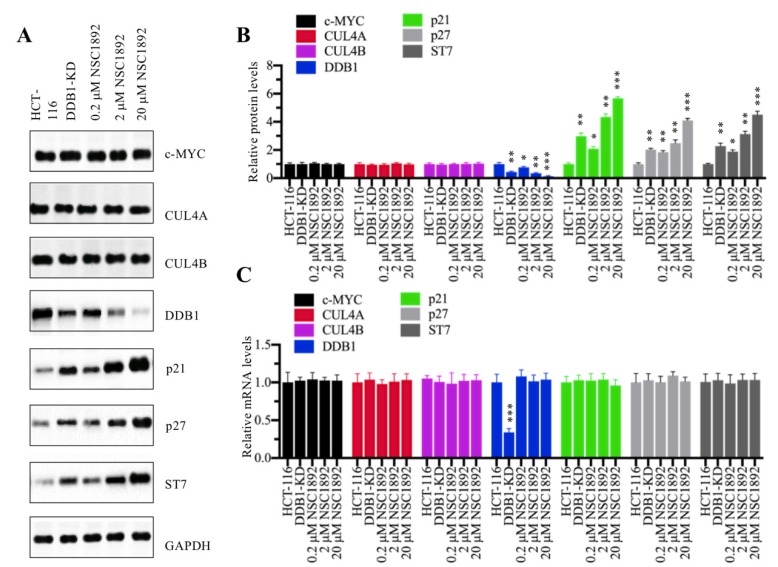
** NSC1892 treatment resulted in the degradation of DDB1 and the accumulation of ST7. (A)** NSC1892 treatment caused DDB1 degradation and ST7 accumulation. HCT-116 cells were treated with different concentrations (0, 0.2, 2, and 20 μM) of NSC1892 for 6 hrs, respectively. The untreated DDB1-KD cells were used as a control. The protein levels of c-MYC, CUL4A, CUL4B, DDB1, ST7, p21 and p27 were determined by western blotting. GAPDH was used as a loading control. **(B)** Quantification of protein levels. The protein levels of c-MYC, CUL4A, CUL4B, DDB1, ST7, p21 and p27 in (A) were quantified using Image J software, and their relative levels in each treatment were normalized to GAPDH. *P < 0.05, ^**^P <0.01 and ***P < 0.001. **(C)** NSC1892 treatment did not change the mRNA levels of *DDB1* and *ST7*. The same cells used in (A) were used for RNA isolation, followed by qRT-PCR analyses to examine mRNA levels of *c-MYC*, *CUL4A*, *CUL4B*, *DDB1*, *ST7*,* p21* and *p27*. ***P < 0.001.

We next sought to examine molecular changes of CRL4^DACF4^ E3 ligases and their substrate ST7 in the treatment of NSC1892. Accordingly, we knocked down *DDB1* in HCT-116 cells to generate a DDB1-KD cell line and then used it as a control to determine the effects of different concentrations of NSC1892 (0, 0.2, 2 and 20 μM) on the protein levels of c-MYC, CUL4A, CUL4B, DDB1 and ST7. Meanwhile, we also detected two known CRL4 E3 ligase substrates p21 (also known as CDKN1A, cyclin-dependent kinase inhibitor 1A) and p27 (also known as CDKN1B). As shown in Figure 3A, we did not observe significant changes of c-MYC, CUL4A and CUL4B protein levels in the conditions of NSC1892 treatments. However, we identified that DDB1 was gradually decreased with the increase of NSC1892 concentrations (Figure 3A). In contrast, the protein levels of ST7, p21, and p27 were gradually accumulated with the increase of NSC1892 concentrations (Figure 3A). The statistical data indicated that treatments with 0.2, 2 and 20 μM NSC1892 caused ~45%, ~70% and ~90% reduction in DDB1 protein level, respectively (Figure 3B), while the same concentrations of NSC1892 resulted in ~2.7, ~4.4 and 6.1-fold increase in ST7 protein level, ~2.1, ~2.9 and 4.1-fold increase in p21 protein level, and ~1.9, ~3.2 and 4.5-fold increase in p27 protein level, respectively (Figure 3B). The protein levels of DDB1, ST7, p21, and p27 in DDB1-KD cells were similar to that in 2 μM NSC1892-treated cells (Figures 3A and 3B). To determine if NSC1892 treatment affected *DDB1* and *ST7* mRNA levels, we carried out qRT-PCR analyses to measure mRNA levels of CRL4^DACF4^ components, *ST7*, *p21* and *p27*. Our results indicated that NSC1892 treatment was not able to change their mRNA levels (Figure 3C). These results suggested that NSC1892 disrupted the CUL4A-DDB1 interaction and caused the degradation of DDB1. The impairment of CRL4 E3 ligases might decrease the ubiquitination of ST7, p21 and p27, thereby leading to their accumulation. To further determine the *in vivo* effect of NSC1892 on the cullin proteins, we also examined protein levels of other cullin family members and the neddylation of CUL4A/4B in NSC1892-treated cells. As shown in Supplementary Figures 4A and 4B, we did not observe a significant difference in the protein levels of cullin members and the neddylation of CUL4A/4B in NSC1892-treated cells compared to untreated cells.

### NSC1892 treatment decreased the CUL4-DDB1 interaction and ST7 ubiquitination level

**Figure 4:**
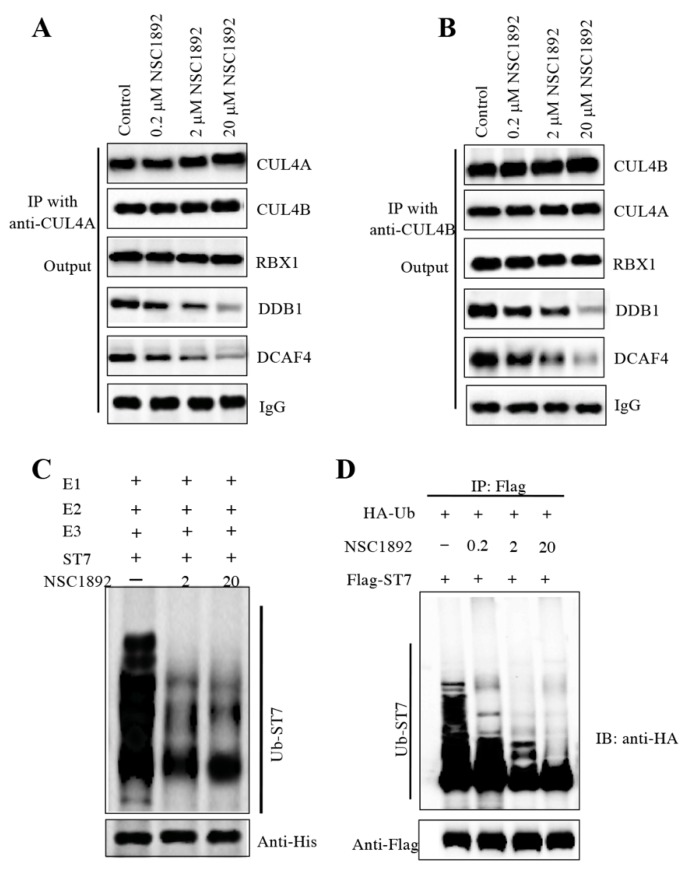
** NSC1892 treatment inhibited the ubiquitination of ST7. (A** and** B)** NSC1892 treatment decreased the CUL4A-DDB1 and CUL4B-DDB1 interactions. HCT-116 cells were treated with different concentrations (0, 0.2, 2, and 20 μM) of NSC1892 for 6 hrs, respectively. Cells were then applied to immunoprecipitation assays using anti-CUL4A **(A)** and anti-CUL4B **(B)** antibodies, respectively. The output protein complexes were used to detect protein levels of CUL4A, CUL4B, RBX1, DDB1 and DCAF4. IgG was used as a loading control.** (C)** NSC1892 treatment inhibited the ubiquitination of ST7 *in vitro*. The purified His-ST7 protein was incubated with E1, E2, and E3 (Flag-CUL4A-associated complex) in a ubiquitination reaction buffer containing different concentrations (0, 2, and 20 μM) of NSC1892, followed by immunoblotting for ST7. The loading level of His-ST7 was examined using an anti-His antibody. **(D)** NSC1892 treatment inhibited the ubiquitination of ST7 *in vivo*. HCT-116 cells under 80% of confluence were cotransfected with pCDNA3-3×HA-ubiquitin and pCDNA3-2×Flag-ST7. After incubation for another 48 hrs, cells were treated with different concentrations (0, 0.2, 2, and 20 μM) of NSC1892 for 6 hrs. Cells were immunoprecipitated with an anti-Flag antibody and the ubiquitination of ST7 was detected using an anti-HA antibody. The loading level of ST7 was examined using an anti-Flag antibody.

Human CUL4A and CUL4B share over 80% amino acid sequence identity 14-19. Since NSC1892 could disrupt the CUL4A-DDB1 interaction, we assumed that it also could inhibit the CUL4B-DDB1 interaction. To verify this hypothesis, we performed immunoprecipitation assays using both anti-CUL4A and anti-CUL4B in cells treated with 0, 0.2, 2 and 20 μM NSC1892. As shown in Figure 4A, the same amounts of CUL4A in cells treated with different concentrations of NSC1892 pulled down similar amounts of CUL4B and RBX1. However, the immunoprecipitated DDB1 and DCAF4 were gradually decreased with the increase of NSC1892 concentrations (Figure 4A). These results suggested that NSC1892 disrupted the CUL4A-DDB1 interaction. Similarly, we also observed that the same amounts of CUL4B pulled down similar amounts of CUL4A and RBX1 but decreased amounts of DDB1 and DCAF4 levels in NSC1892-treated cells (Figure 4B). These results suggested that NSC1892 not only disrupted the CUL4A-DDB1 interaction, but also inhibited the CUL4B-DDB1 interaction. To further verify that NSC1892 could impair the CUL4-DDB1 interaction, we also performed an *in vitro* pulldown assay using the recombinant GST-CUL4A and His-DDB1 proteins. Accordingly, we initially incubated these two proteins to assemble a complex. After treating it with different concentrations of NSC1892 (0, 2 and 20 μM), proteins were pulled down with Ni-NTA resin. The results showed that NSC1892 treatments significantly decreased the GST-CUL4A protein levels pulled down by the same amounts of His-DDB1 (Supplementary Figure 5).

The disruption of CUL4-DDB1 interaction by NSC1892 should affect the assembly of CRL4^DCAF4^ complex and thus inhibit the ubiquitination of ST7. To test this hypothesis, we performed an *in vitro* assay to examine the ubiquitination level of ST7 in the treatments of NSC1892 (0, 2 and 20 μM). Our result indicated that the ubiquitination level of ST7 was gradually decreased with the increase of NSC1892 concentrations (Figure 4C). In addition, we also evaluated the *in vivo* effect of NSC1892 on ST7 ubiquitination. Accordingly, we cotransfected pCDNA3-2×Flag-ST7 and pCDNA3-3×HA-Ubiquitin into HCT-116 cells, followed by treating cells with 0, 0.2, 2 and 20 μM NSC1892, respectively*.* The ubiquitination analysis result indicated that the ubiquitination level of ST7 was significantly decreased in NSC1892-treated cells compared to untreated cells (Figure 4D)*.* Given that K48-linked polyubiquitin chains are involved in proteasomal degradation, we next sought to determine if NSC1892 could affect K48-linked polyubiquitination of ST7. For this purpose, we cotransfected pCDNA3-2×Flag-ST7 and pCDNA3-3×HA-Ubiquitin-K48R into HCT-116 cells, followed by treating cells with 0, 0.2, 2 and 20 μM NSC1892, respectively*.* The ubiquitination analysis result indicated that the K48-linked polyubiquitination of ST7 was significantly decreased in NSC1892-treated cells compared to untreated cells (Supplementary Figure 6).

### NSC1892 inhibited the growth of other *CUL4A*- or *CUL4B*-overexpression tumor cells

**Figure 5:**
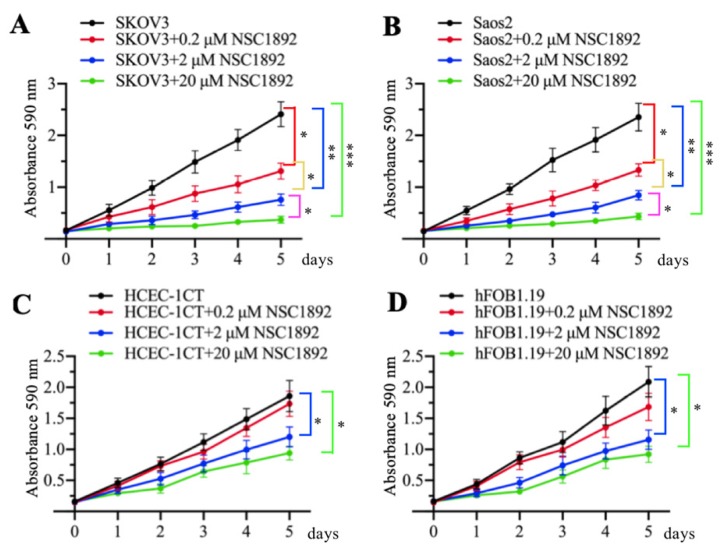
** NSC1892 treatment also caused the growth inhibition of other cancer cells with *CUL4A* or *CUL4B* overexpression. (A** and** B)** NSC1892 treatment inhibited cell proliferation in *ovarian and osteosarcoma cell lines. A CUL4A-overexpression ovarian cell line SKOV3 **(A)** and a CUL4B-overexpression osteosarcoma cell line Saos2 **(B)*** were treated with different concentrations (0, 0.2, 2, and 20 μM) of NSC1892 for 5 days. Cells viability was measured each day using an MTT assay. ^*^P <0.05, ^**^P <0.01 and ^***^P <0.001. (C and D) High dose of NSC1892 inhibited cell proliferation in *noncancerous cells.* A human colon epithelial cell line HCEC-1CT (C) and an osteoblast cell line hFOB1.19 (D) were treated with different concentrations (0, 0.2, 2, and 20 μM) of NSC1892 for 5 days. Cells viability was measured each day using an MTT assay. ^*^P <0.05.

As mentioned early, *CUL4A* and *CUL4B* are overexpressed in many cancer types 14-19. Since NSC1892 specifically targeted the CUL4A/4B-DDB1 interactions, we speculated that it should also be effective to inhibit the cell growth of other *CUL4A*- or *CUL4B*-overexpression tumor cells, not only CRC cells. To validate this hypothesis, we selected a CUL4A-overexpression ovarian cell line SKOV3 and a CUL4B-overexpression osteosarcoma cell line Saos2 and evaluated their cell proliferation abilities in the conditions of 0, 0.2, 2 and 20 μM NSC1892 treatments, respectively. As shown in Figures 5A and 5B, 0.2, 2 and 20 μM NSC1892 treatments resulted in ~50%, ~75% and ~90% reduction in cell proliferation compared to non-treatment. These results demonstrated that NSC1892 was a promising compound that inhibited the growth of CUL4A- and CUL4B-overexpression cancer cells. A very critical issue for the clinical trials of chemotherapeutic drugs is their toxicity to noncancerous cells. An ideal chemotherapy drug should specifically kill cancer cells without causing significant damage to normal cells. Thus, we next sought to determine the cytotoxicity of NSC1892 to noncancerous cells. Accordingly, we treated a human colon epithelial cell line HCEC-1CT and an osteoblast cell line hFOB1.19 with 0, 0.2, 2 and 20 μM NSC1892 and then evaluated cell proliferation. Our results showed that NSC1892 had much weaker cytotoxicity to these two noncancerous cells (Figures 5C and 5D). There was no significant difference between 0.2 μM NSC1892-treated cells and controls, while treatments with 2 and 20 μM NSC1892 resulted in ~25-30% and ~40-45% decrease in cell proliferation compared to non-treatment (Figures 5C and 5D). These results suggested that NSC1892 showed much weaker cytotoxicity to noncancerous cells. In addition, we also examined the protein levels of CRL4^DCAF4^ components and ST7 in these NSC1892-treated cells. Consistent with the results in HCT-116 cells, we also did not observe significant changes of c-MYC, CUL4A and CUL4B protein levels when cells were treated with different concentrations of NSC1892 (Supplementary Figure 7). However, DDB1 was degraded in a dose-dependent manner, while ST7 was accumulated with the increase of NSC1892 concentrations in all four cell lines (Supplementary Figure 7). These results indicated that NSC1892 inhibited cell growth by disrupting the interactions of CUL4A/4B-DDB1.

### NSC1892 significantly inhibited tumor growth *in vivo*

**Figure 6:**
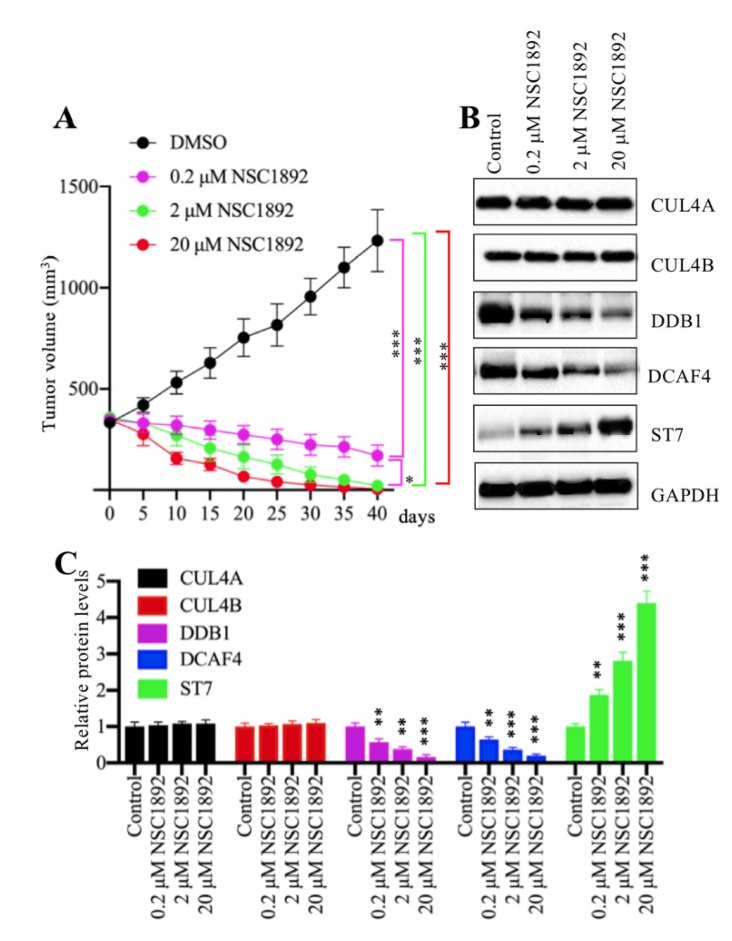
** NSC1892 inhibited tumor growth *in vivo*. (A)** NSC1892 inhibited tumor growth *in vivo*. HCT-116 cells were injected into nude mice to generate tumors. After 25 days, mice harboring ~400 mm^3^ tumor volumes were randomly assigned to four groups (n = 5 in each group). These four-group mice were then injected with DMSO 0.2 μM, 2.0 μM or 20 μM NSC1892 twice a week, respectively. Tumor volumes were monitored for another 40 days. *P < 0.05 and ***P < 0.001. **(B** and** C)** NSC1892 treatment impaired the assembly of CRL4^DACF4^ complexes *in vivo*. Tumors from mice treated with different concentrations of NSC1892 were used to extract total proteins, followed by immunoblots to examine the protein levels of CUL4A, CUL4B, DDB1, DCAF4 and ST7. GAPDH was used as a loading control **(B)**. The protein levels in (B) were quantified and normalized to GAPDH and shown in **(C)**. **P < 0.01 and ***P < 0.001.

Our *in vitro* results showed that NSC1892 significantly inhibited CRC cell proliferation, colony formation and cell invasion. We speculated that it should be effective *in vivo*. Thus, we injected HCT-116 cells into mice to generate tumors. After 25 days, mice harboring ~400 mm^3^ tumor volumes were randomly assigned to four groups (n = 5). The first group was set as control and only injected with DMSO. The other three groups were injected with 0.2 μM, 2.0 μM or 20 μM NSC1892 twice a week, respectively. Tumor volumes were monitored for another 40 days, and the results indicated tumor volumes in mice injected with NSC1892 were significantly decreased compared to mice injected with DMSO (Figure 6A). A very interesting phenomenon was that the tumors not only stopped growing, but their volumes were constantly shrinking after the injection of NSC1892. In 2 μM- and 20 μM-treated mice, the tumors almost completely disappeared on the 40^th^ day. This interesting result indicated that NSC1892 was also very active in mice. Moreover, we also examined protein levels of CRL4^DCAF4^ complex and its substrate ST7 in tumors from mice treated with or without NSC1892 treatment. Consistent with the *in vitro* results, we also observed treatments with different concentrations of NSC1892 could not change CUL4A and CUL4B protein levels (Figure 6B). However, the protein levels of DDB1 and DCAF4 were gradually decreased while ST7 was constantly increased in mice treated with 0.2 μM, 2.0 μM or 20 μM NSC1892 (Figure 6B). The quantified protein levels showed that treatments with 0.2, 2 and 20 μM NSC1892 caused ~40-50%, ~60-70% and ~80-90% reduction in DDB1 and DCAF4 protein levels, while the same concentrations of NSC1892 resulted in ~1.9, ~3.1 and 4.3-fold increase in the ST7 protein level, respectively, in comparison to control group mice (Figure 6C). These results suggested that NSC1892 was effective *in vivo* through disrupting the CUL4A/4B-DDB1 interactions, causing the degradation of DDB1 and the instability of CRL4^DACF4^ complex, and eventually leading to the accumulation of ST7.

## Discussion

**Figure 7:**
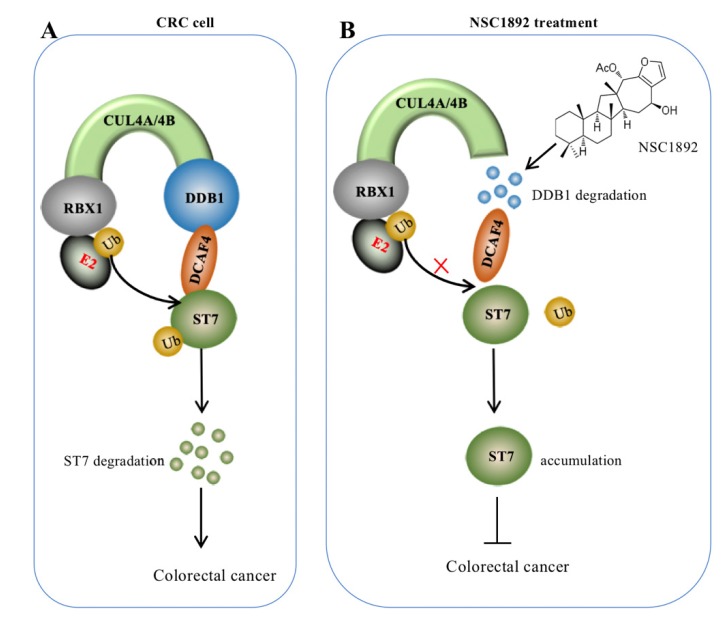
** A schematic diagram of NSC1892 action. (A)** A schematic diagram of ST7 degraded by the CRL4^DCAF4^ E3 ligases. In CRC cells, both CUL4A and CUL4B are amplified. They function as a platform to recruit RBX1, DDB1 and DCAF4 to assemble CRL4^DCAF4^ E3 ligases, which specifically ubiquitinate ST7 and cause its degradation. **(B)** A schematic diagram of NSC1892 action. NSC1892 specifically blocks the CUL4A/4B-DDB1 interactions and causes the degradation of DDB1. The degraded DDB1 impairs the assembly of CRL4^DCAF4^ E3 ligases, which decreases the ubiquitination of ST7 and causes its accumulation. The accumulated ST7 functions as a tumor suppressor to prevent cancer cell growth.

Overexpression of *CUL4A* or *CUL4B* is a very common sign in many types of cancers in which CUL4A or CUL4B proteins conservatively interacts with DDB1 to recruit other proteins such as RBXs and DACFs, assembling CRL4 E3 ligases 14-19. The activated CRL4 E3 ligases recognize different substrates such as tumor suppressors ST7 and PTEN (phosphatase and tensin homolog deleted on chromosome 10) 10, 14, and cell cycle regulator p21 and p27 13, 24. The hyperubiquitination and degradation of these substrates result in the tumorigenesis 10, 14-19, 24. The interaction between CUL4A/4B and DDB1 is required for the assembly of all CRL4 E3 ligases 10, 14-19, 24. Thus, disruption of the interactions of CUL4A/4B-DDB1 could be an effective strategy to treat cancer with *CUL4A/4B*-overexpression. In this study, we developed an AlphaScreen HTS method to identify compounds that were able to disrupt the CUL4A-DDB1 interaction. Using this method, we obtained an effective compound NSC1892, which showed a strong ability to inhibit the interactions between CUL4A/4B and DDB1 and caused the degradation of DDB1. The decreased DDB1 could not recruit DCAF4 efficiently and caused the impairment of CRL4^DCAF4^ E3 ligases, which resulted in the repression of ST7 ubiquitination. The accumulated ST7 functioned as a tumor suppressor to inhibit the growth of cancer cells (Figure 7).

In our experiments, we validated that NSC1892 was not only effective in CRC cells, but also showed strong inhibitory effects on other cells with *CUL4A* or *CUL4B* overexpression. However, we found that high concentrations of NSC1892 also showed cytotoxicity in noncancerous cells (Figures 5C and 5D). Although the cytotoxicity was weaker compared to cancer cells (Figure 5), it might also limit its application in clinical trials. Thus, we are making some chemical modifications to this compound in order to obtain some less cytotoxic derivatives. Recently, Chen and colleagues also discovered a naturally-sourced small molecule TSC01131 using a yeast HTS method 10. Their results showed that TSC01131specifically inhibited the CUL4B-DDB1 interaction in osteosarcoma cells, and TSC01131 could also inhibit osteosarcoma cell growth 10. Although it seems like that the functional mechanisms of NSC1892 and TSC01131 are similar, we do not find a similarity in the chemical structures of these two compounds. Unfortunately, TSC01131 is not commercially available, so we cannot use it as a control in our experiments. In addition, we also did not use any commercial chemotherapeutic medicine as a positive control to compare their cytotoxicities in our experiments, which limited our understanding and comparison of the efficacy of NSC1892 with commercial chemotherapeutic medicines.

After treatment with NSC1892, we found that the protein levels of CUL4A/4B were not changed, but it caused a decrease in the DDB1 protein level. This phenomenon is very interesting, and we speculate that there may be two possible mechanisms. One is that NSC1892 treatment directly causes DDB1 degradation, which makes DDB1 unable to interact with CUL4A/4B. Another possibility is that NSC1892 only disrupts the CUL4A/4B-DDB1 interactions and prevents the binding of these two proteins. The disassociated DDB1 may be modified by other proteins, causing its degradation. Two well-known protein modifications are phosphorylation and ubiquitination 25, 26. Currently, there are no commercially available antibodies for detecting DDB1 phosphorylation and ubiquitination. Moreover, we also analyzed the protein sequence of DDB1 and tried to verify the second possibility using DDB1 mutations. However, we found that DDB1 protein sequence contains 152 Ser (S) and Thr (T), and 57 Lys (K) (Supplementary Figures 8 and 9). These amino acids are widely distributed, and it requires a lot of work to complete the verification. We are currently trying to generate some DDB1 point mutations, and we will carry out *in vitro* biochemistry assays to examine if NSC1892 treatment would cause degradation of DDB1 point mutations.

In conclusion, our study discovered NSC1892 could block the CUL4A/4B-DDB1 interactions, causing DDB1 degradation and the impaired assembly of CRL4^DCAF4^ complexes. The decreased activities CRL4^DCAF4^ E3 ligases resulted in repression of ST7 ubiquitination, leading to its accumulation, which inhibited tumor cell growth *in vitro* and *in vivo*.

## Supplementary Material

Supplementary figures and table.
