# Supplementary material for: Small molecule NSC1892 targets the CUL4A/4B-DDB1 interactions and causes impairment of CRL4DCAF4 E3 ligases to inhibit colorectal cancer cell growth
Source: Int J Biol Sci. 2020 Feb 4;16(6):1059–70. doi: 10.7150/ijbs.40235 (PMC7053343; doi:10.7150/ijbs.40235)
Supplement: Supplementary file 1 — Supplementary figures and table. [file ijbsv16p1059s1.pdf]

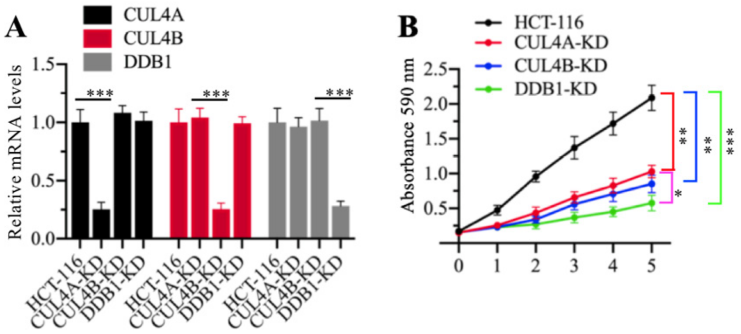

Supplementary Figure 1. Knockdown of *CUL4A/4B* and *DDB1* significantly inhibited tumor cell proliferation.

(A) The mRNA levels of *CUL4A/4B* and *DDB1* in their corresponding knockdown cells. HCT-116 cells were transfected with pLKO.1, shCUL4A (CUL4A-KD), shCUL4B (CUL4B-KD), and shDDB1 (DDB1-KD), respectively. The obtained knockdown cells were subjected to RNA isolation and qRT-PCR analyses to examine mRNA levels of *CUL4A*, *CUL4B* and *DDB1*. ***$P < 0.001$. **(B)** Knockdown of *CUL4A/4B* and *DDB1* decreased cell proliferation. Cells used in (A) were used to examine cell viability at 4 h (0 days), 1, 2, 3, 4, and 5 days by an MTT assay with absorbance measurement at 590 nm. *$P < 0.05$, **$P < 0.01$ and ***$P < 0.001$.

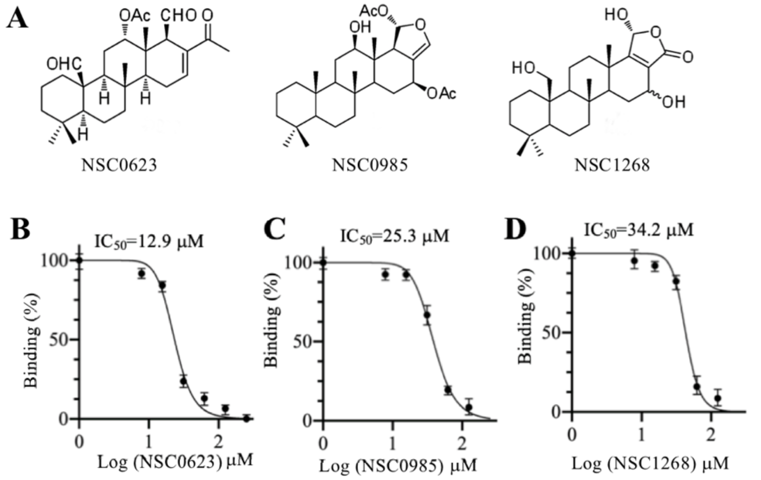

Supplementary Figure 2. Identified small molecules using AlphaScreen system.

(A) The chemical structure of small molecules. The chemical structures of three other small molecules (NSC0623, NSC0985 and NSC1268) that disrupt the CUL4A-DDB1 interaction. **(B-D)** Different effects of small molecules on the CUL4A-DDB1 interaction. A secondary AlphaScreen assay was performed to determine the inhibitory effect of NSC0623, NSC0985 and NSC1268 on the CUL4A-DDB1 interaction.

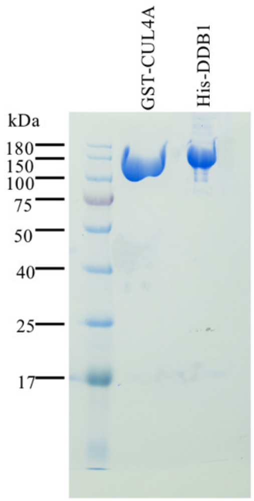

Supplementary Figure 3. The purified GST-CUL4A and His-DDB1 proteins.

The pET28a-DDB1 and pGEX-6P-1-CUL4A plasmids were transformed into an *Escherichia coli* strain BL21 (DE3.0), respectively. The positive colonies were grown in liquid lysogeny broth (LB) medium containing antibiotics to logarithmic phase. The GST-CUL4A and His-DDB1 proteins were purified using Glutathione Sepharose 4B resin and Ni-NTA resin, respectively. Equal amounts of GST-CUL4A and His-DDB1 proteins were loaded into a 10% SDS-PAGE gel for separation and then stained with Coomassie blue.

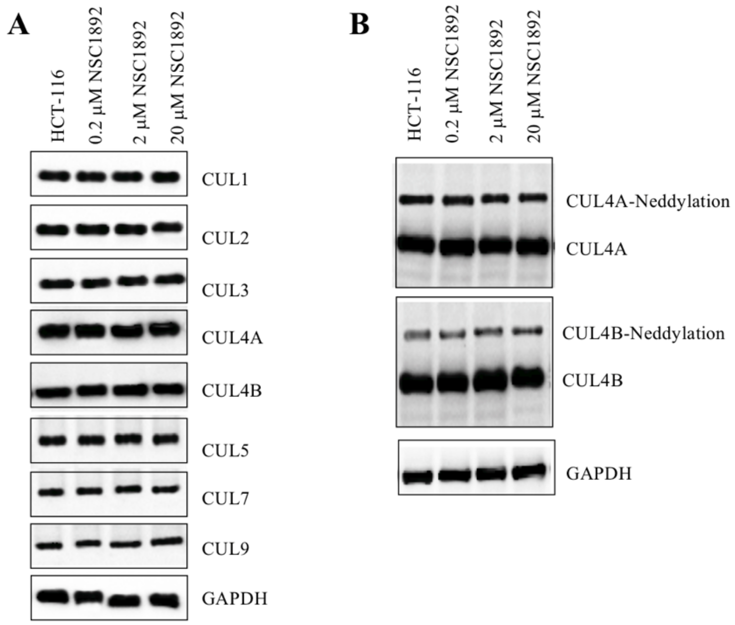

Supplementary Figure 4. NSC1892 could not change the protein levels of cullin members and the neddylation of ST7.

(A) NSC1892 treatment could not change the protein levels of cullin members. The HCT-116 cells were treated with different concentrations (0, 0.2, 2, and 20 μM) of NSC1892 for 6 hrs. The resulting cells were subjected to protein isolation. The protein levels of CUL1, CUL2, CUL3, CUL4A, CUL4B, CUL5, CUL7 and CUL9 were determined by western blotting. GAPDH was used as a loading control. **(B)** NSC1892 treatment could not change the neddylation of ST7. Proteins used in (A) were loaded into a 10% SDS-PAGE for efficient separation (run 2 hrs at 180 V). The neddylation of CUL4A /4B was determined using anti-CUL4A and anti-CUL4B antibodies, respectively.

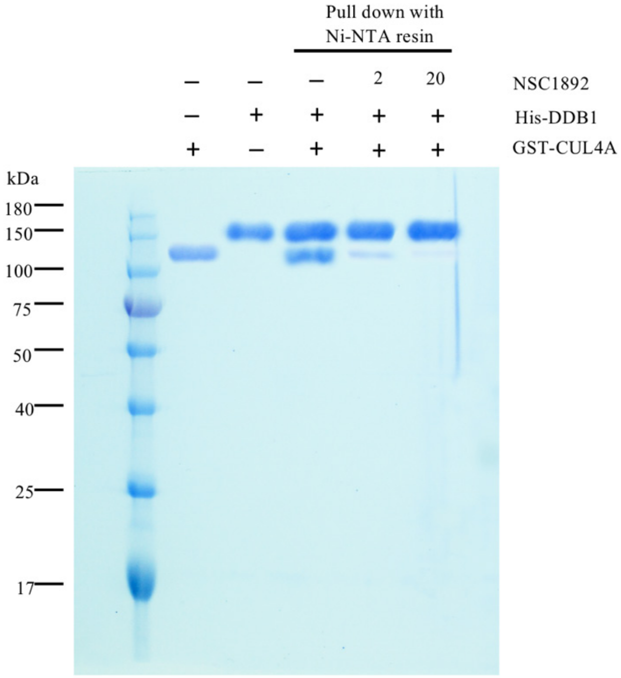

Supplementary Figure 5. NSC1892 decreased the interaction of CUL4A and DDB1 *in vitro*. Equal amounts of GST-CUL4A and His-DDB1 proteins were incubated to assemble a complex at 4°C for 30 min. The CUL4A-DDB1 complex was then treated with DMSO, 2.0 μM or 20 μM NSC1892 at 4°C for 6 hrs, followed by incubating with the Ni-NTA resin at 4°C for 4 hrs. After washing five times, the pulldown proteins were determined by loading into a 10% SDS-PAGE gel and then stained with Coomassie blue.

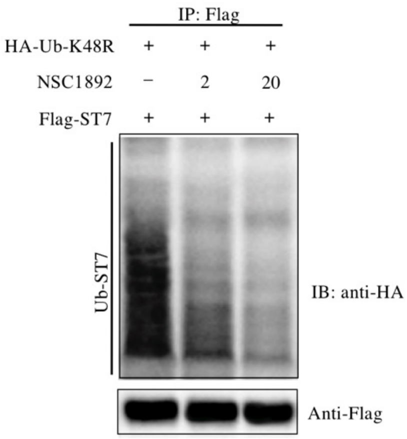

Supplementary Figure 6. NSC1892 inhibited the K48-linked poly-ubiquitination of ST7. HCT-116 cells under 80% confluence were cotransfected with pCDNA3-3×HA-ubiquitin-K48R and pCDNA3-2×Flag-ST7. After incubation for another 48 hrs, cells were treated with different concentrations (0, 0.2, 2, and 20 μM) of NSC1892 for 6 hrs. Cells were immunoprecipitated with an anti-Flag antibody and the ubiquitination of ST7 was detected using an anti-HA antibody. The loading level of ST7 was examined using an anti-Flag antibody.

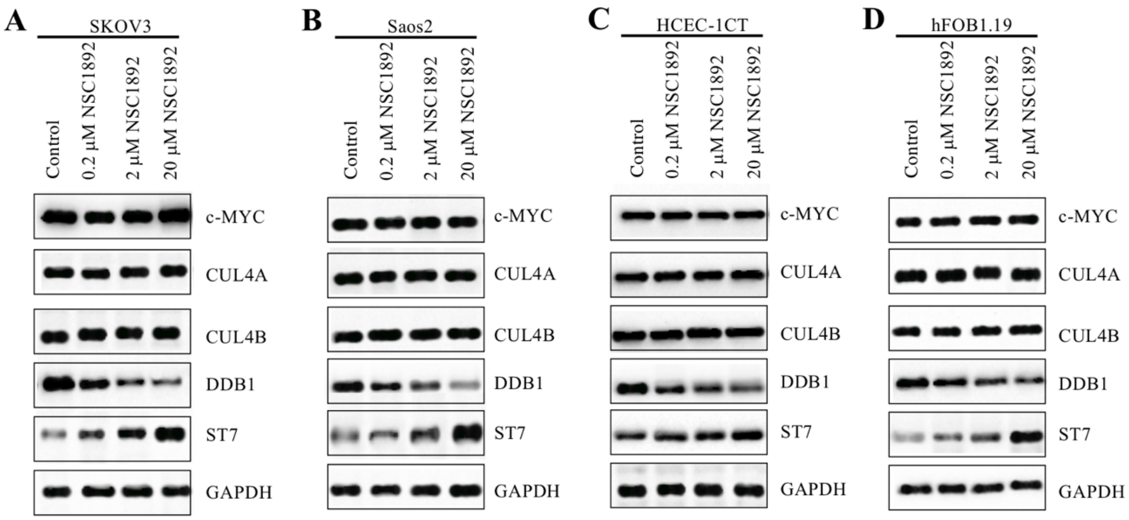

Supplementary Figure 7. NSC1892 degraded DDB1 protein in cancerous and noncancerous cells

SKOV3 **(A)**, Saos2 **(B),** HCEC-1CT **(C)**, and hFOB1.19 **(D)** cells were treated with different concentrations (0, 0.2, 2, and 20 μM) of NSC1892 for 6 hrs, respectively. The protein levels of c-MYC, CUL4A, CUL4B, DDB1, and ST7 were determined by western blotting. GAPDH was used as a loading control.

The Ser/Thr sites of DDB1

```
MSYNYVVTAQKPTAVNGCVTGHFTSAEDLNLLIAKNTRLEIYVVTAEGLRPVKEVGMYGK
IAVMELFRPKGESKDLLFILTAKYNACILEYKQSGESIDIITRAHGNVQDRIGRPSETGI
IGIIDPECRMIGLRLYDGLFKVIPLDRDNKELKAFNIRLEELHVIDVKFLYGCQAPTICF
VYQDPQGRHVKTYEVSLREKEFNKGPWKQENVEAEASMVIAVPEPFGGAIIIGQESITYH
NGDKYLAIAPPIIKQSTIVCHNRVDPNGSRYLLGDMEGRLFMLLLEKEEQMDGTVTLKDL
RVELLGETSIAECLTYLDNGVVFVGSRLGDSQLVKLNVDSNEQGSYVVAMETFTNLGPIV
DMCVVDLERQGQGQLVTCSGAFKEGSLRIIRNGIGIHEHASIDLPGIKGLWPLRSDPNRE
TDDTLVLSFVGQTRVLMLNGEEVEETELMGFVDDQQTFFCGNVAHQQLIQITSASVRLVS
QEPKALVSEWKEPQAKNISVASCNSSQVVVAVGRALYYLQIHPQELRQISHTEMEHEVAC
LDITPLGDSNGLSPLCAIGLWTDISARILKLPSFELLHKEMLGGEIIPRSILMTTFESSH
YLLCALGDGALFYFGLNIETGLLSDRKKVTLGTQPTVLRTFRSLSTTNVFACSDRPTVIY
SSNHKLVFSNVNLKEVNYMCPLNSDGYPDSLALANNSTLTIGTIDEIQKLHIRTVPLYES
PRKICYQEVSQCFGVLSSRIEVQDTSGGTTALRPSASTQALSSSVSSSKLFSSSTAPHET
SFGEEVEVHNLLIIDQHTFEVLHAHQFLQNEYALSLVSCKLGKDPNTYFIVGTAMVYPEE
AEPKQGRIVVFQYSDGKLQTVAEKEVKGAVYSMVEFNGKLLASINSTVRLYEWTTEKELR
TECNHYNNIMALYLKTKGDFILVGDLMRSVLLLAYKPMEGNFEEIARDFNPNWMSAVEIL
DDDNFLGAENAFNLFVCQKDSAATTDEERQHLQEVGLFHLGEFVNVFCHGSLVMQNLGET
STPTQGSVLFGTVNGMIGLVTSLSESWYNLLLDMQNRLNKVIKSVGKIEHSFWRSFHTER
KTEPATGFIDGDLIESFLDISRPKMQEVVANLQYDDGSGMKREATADDLIKVVEELTRIH
```

Supplementary Figure 8. The Ser/Thr sites in the protein sequence of DDB1.

The Ser/Thr sites in the protein sequence of DDB1 were labelled with green and purple colors, respectively.

The K sites of DDB1

```
MSYNYVVTAQKPTAVNGCVTGHFTSAEDLNLLIAKNTRLEIYVVTAEGLRPVKEVGMYGK
IAVMELFRPKGESKDLLFILTAKYNACILEYKQSGESIDIITRAHGNVQDRIGRPSETGI
IGIIDPECRMIGLRLYDGLFKVIPLDRDNKELKAFNIRLEELHVIDVKFLYGCQAPTICF
VYQDPQGRHVKTYEVSLREKEFNKGPWKQENVEAEASMVIAVPEPFGGAIIIGQESITYH
NGDKYLAIAPPIIKQSTIVCHNRVDPNGSRYLLGDMEGRLFMLLLEKEEQMDGTVTLKDL
RVELLGETSIAECLTYLDNGVVFVGSRLGDSQLVKLNVDSNEQGSYVVAMETFTNLGPIV
DMCVVDLERQGQGQLVTCSGAFKEGSLRIIRNGIGIHEHASIDLPGIKGLWPLRSDPNRE
TDDTLVLSFVGQTRVLMLNGEEVEETELMGFVDDQQTFFCGNVAHQQLIQITSASVRLVS
QEPKALVSEWKEPQAKNISVASCNSSQVVVAVGRALYYLQIHPQELRQISHTEMEHEVAC
LDITPLGDSNGLSPLCAIGLWTDISARILKLPSFELLHKEMLGGEIIPRSILMTTFESSH
YLLCALGDGALFYFGLNIETGLLSDRKKVTLGTQPTVLRTFRSLSTTNVFACSDRPTVIY
SSNHKLVFSNVNLKEVNYMCPLNSDGYPDSLALANNSTLTIGTIDEIQKLHIRTVPLYES
PRKICYQEVSQCFGVLSSRIEVQDTSGGTTALRPSASTQALSSSVSSSKLFSSSTAPHET
SFGEEVEVHNLLIIDQHTFEVLHAHQFLQNEYALSLVSCKLGKDPNTYFIVGTAMVYPEE
AEPKQGRIVVFQYSDGKLQTVAEKEVKGAVYSMVEFNGKLLASINSTVRLYEWTTEKELR
TECNHYNNIMALYLKTKGDFILVGDLMRSVLLLAYKPMEGNFEEIARDFNPNWMSAVEIL
DDDNFLGAENAFNLFVCQKDSAATTDEERQHLQEVGLFHLGEFVNVFCHGSLVMQNLGET
STPTQGSVLFGTVNGMIGLVTSLSESWYNLLLDMQNRLNKVIKSVGKIEHSFWRSFHTER
KTEPATGFIDGDLIESFLDISRPKMQEVVANLQYDDGSGMKREATADDLIKVVEELTRIH
```

Supplementary Figure 9. The Lys sites in the protein sequence of DDB1.

The Lys (K) sites in the protein sequence of DDB1 were labelled with light blue color.

Supplementary Table-1. Primers used for qRT-PCR analyzes

Gene	Forward Primers	Reverse primers
CUL4A	5’- ACCATTGATGGAATCCTACTGC-3’	5’- ACCTTTGGCCTTCGGCAGCATA-3’
CUL4B	5’-ACTTCGTGCAGGCAACAAAGA -3’	5’- CAGCATCTACAGATGCACTCT-3’
c-Myc	5’-ACCACCAGCAGCGACTCT-3’	5’- GCGTAGTTGTGCTGATGTGT-3’
DDB1	5’- TAGAGATCTATGTGGTCAC-3’	5’- ATGCAGGCATTGTACTTCGCTG-3’
ST7	5’-GTCTCAGTAAGCCACTTGC-3’	5’- GTCATAGTAAGTAAGAGGCT-3’
p21	5’-CCTCAGGCAGCTCAAGCAGCG-3’	5’-AGGGGCCAGTGTCTCCCTCCT-3’
p27	5’-AACTGACGTGGAGCGGGGTAT-3’	5’-AATGAAGTATCAGCTGTCTCT-3’
β-Actin	5’- AGAGCTACGAGCTGCCTGAC-3’	5’- AGCACTGTGTTGGCGTACAG -3’
